# Transplanted Neural Stem Cells Modulate Regulatory T, γδ T Cells and Corresponding Cytokines after Intracerebral Hemorrhage in Rats

**DOI:** 10.3390/ijms15034431

**Published:** 2014-03-13

**Authors:** Lu Gao, Qin Lu, Li-Jie Huang, Lin-Hui Ruan, Jian-Jing Yang, Wei-Long Huang, Wei-Shan ZhuGe, Yong-Liang Zhang, Biao Fu, Kun-Lin Jin, Qi-Chuan ZhuGe

**Affiliations:** 1 Department of Neurosurgery, First Affiliated Hospital, Wenzhou Medical University, Wenzhou 325000, Zhejiang, China; E-Mails: gl2008123@126.com (L.G.); happycatlq1001@gmail.com (Q.L.); lijiehuangwy@gmail.com (L.-J.H.); rlh1232@hotmail.com (L.-H.R.); jianjingyang11@hotmail.com (J.-J.Y.); weilongh84@hotmail.com (W.-L.H.); drzhangyl33@gmail.com (Y.-L.Z.); drxf39@gmail.com (B.F.); 2 Zhejiang Provincial Key Laboratory of Aging and Neurological Disorder Research, First Affiliated Hospital, Wenzhou Medical University, Wenzhou 325000, Zhejiang, China; E-Mails: cupidjiro@gmail.com (W.-S.Z.G.); Kunlin.Jin@unthsc.edu (K.-L.J.); 3 Department of Pharmacology and Neuroscience, University of North Texas Health Science Center, Fort Worth, TX 76107, USA

**Keywords:** ICH, transplantation, NSCs, T lymphocyte subpopulations, immunomodulation

## Abstract

The immune system, particularly T lymphocytes and cytokines, has been implicated in the progression of brain injury after intracerebral hemorrhage (ICH). Although studies have shown that transplanted neural stem cells (NSCs) protect the central nervous system (CNS) from inflammatory damage, their effects on subpopulations of T lymphocytes and their corresponding cytokines are largely unexplored. Here, rats were subjected to ICH and NSCs were intracerebrally injected at 3 h after ICH. The profiles of subpopulations of T cells in the brain and peripheral blood were analyzed by flow cytometry. We found that regulatory T (Treg) cells in the brain and peripheral blood were increased, but γδT cells (gamma delta T cells) were decreased, along with increased anti-inflammatory cytokines (IL-4, IL-10 and TGF-β) and decreased pro-inflammatory cytokines (IL-6, and IFN-γ), compared to the vehicle-treated control. Our data suggest that transplanted NSCs protect brain injury after ICH via modulation of Treg and γδT cell infiltration and anti- and pro-inflammatory cytokine release.

## 1. Introduction

Intracerebral hemorrhage (ICH) is a subtype of stroke with the highest rates of mortality and morbidity. The 30-day mortality rate is 45% and most survivors are left with severe neurological disabilities after ICH [1]. The primary brain damage after ICH is compression of the brain parenchyma caused by rapid hematoma formation [2,3]. The secondary injury after ICH is mainly due to inflammatory response, which includes inflammatory cytokines, complement components, microglial activation, and immune cells infiltration [4,5].

Both innate and adaptive immune systems have been proven to participate in the progression of cellular injury after stroke [6]. For example, the αβT-lymphocyte, including both CD4^+^ or CD8^+^ T-cell, and γδT cells (gamma delta T cells) can infiltrate the brain at the acute phase of ischemic stroke [7]. Previous studies suggested that most T lymphocytes play a detrimental role in brain ischemic injury [8–10]. However, recent studies indicate that some T cell subsets, such as regulatory T (Treg) cells, a key cerebroprotective immunomodulators, ameliorated brain injury after ischemic stroke [11,12].

Transplantation of NSCs has been proposed as a promising strategy for promoting functional recovery after ICH. Previous studies show that the mechanisms underlying neuroprotection mediated by transplanted NSCs is due to directly cell replacement [13,14]. Recent evidence indicates that the transplanted NSCs protect the CNS from inflammatory via a “bystander” mechanism rather than direct cell replacement [15,16]. The NSCs-mediated immunomodulation of anti-inflammatory effect after transplantation has been documented on several CNS diseases, including acute and chronic experimental autoimmune encephalomyelitis (EAE) [17,18] and neurodegenerative conditions [19]. *In vivo* and *in vitro* studies have also suggested that NSCs inhibit T cells proliferation and promote their apoptosis [17,20,21]. These findings suggest that the transplanted NSCs may modulate T cells and reduce cerebral inflammation after ICH.

Thus, in this study, we investigated whether NSCs would affect the subpopulations of T cells and their corresponding cytokines in the cerebral and peripheral blood after ICH.

## 2. Results

### 2.1. T Lymphocyte Profiles in the Brain and Peripheral Blood

**Figure 1.**
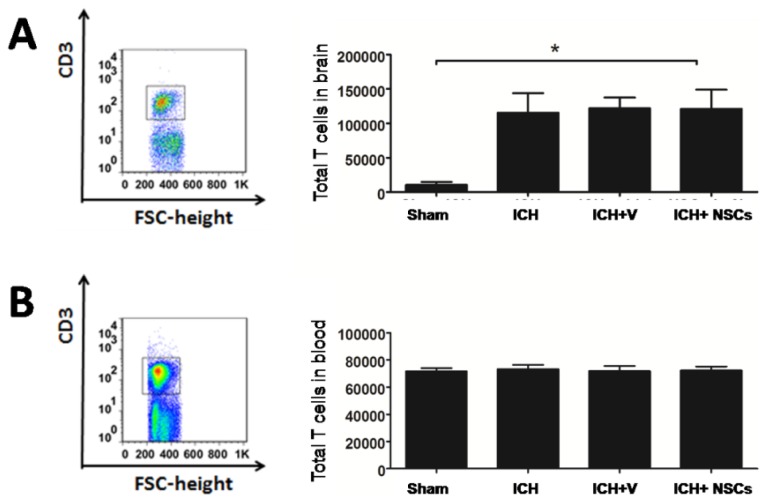
Total numbers of T lymphocyte cells in the brain and peripheral blood. Total numbers of T lymphocytes in the brain were significantly increased in intracerebral hemorrhage (ICH), vehicle-treated (ICH + V) and neural stem cells (NSCs)-treated groups (ICH + NSCs), compared to sham-operated group (Sham) (**A**); there was no difference of total numbers of T lymphocytes of each group in peripheral blood in ICH, vehicle-treated (ICH + V) and NSCs-treated groups (ICH + NSCs), compared to sham-operated group (Sham) (**B**), (*n* = 8 in each group). * *p* < 0.05 *versus* sham ICH.

As a study showed that T lymphocytes in the brain was significantly increased 3 days after ischemic stroke [22], we asked whether the infiltration of T lymphocytes in the brain was also altered after ICH. As shown in Figure 1A, the absolute numbers of T lymphocytes were significantly increased in the brain after ICH, compared to sham-operated rats. However, no difference of T lymphocytes in the peripheral blood was found between ICH and sham-operated rats (Figure 1B).

### 2.2. Increased T Lymphocyte Subpopulations after ICH

**Figure 2.**
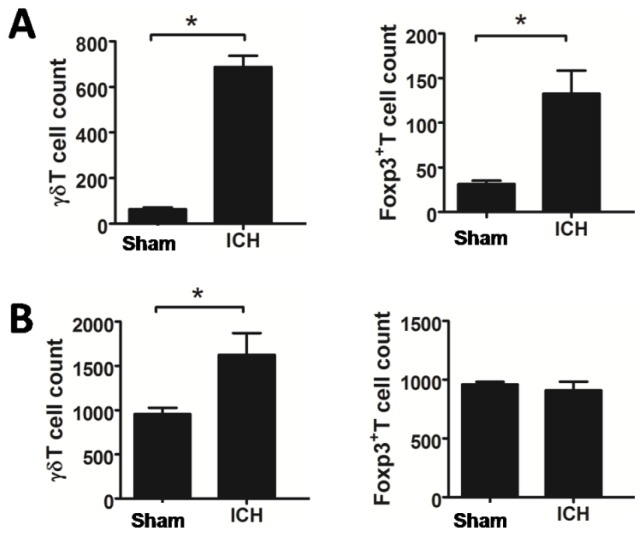
Increased absolute numbers of γδT and Treg cells in the brain and peripheral blood after ICH. The absolute numbers of γδT and Treg cells were increased significantly in the brain (**A**) and peripheral blood; (**B**) after ICH. (*n* = 8 in each group) * *p* < 0.05 *versus* sham ICH.

Recent studies have revealed that γδT cells contribute to brain injury of ischemic stroke, whereas Treg cells are cerebroprotective in acute ischemic stroke. γδT and Treg cells in the brain and peripheral blood were thus examined 3 days after ICH. As shown in Figure 2A, the absolute numbers of γδT and Treg cells were increased significantly in the brain after ICH. γδT lymphocytes were also increased in the peripheral blood, compared to control. However, there was no difference of Treg cells in the peripheral blood between ICH and sham-operated rats (Figure 2B).

### 2.3. Transplanted NSCs Improve Neurological Function Recovery after ICH

**Figure 3.**
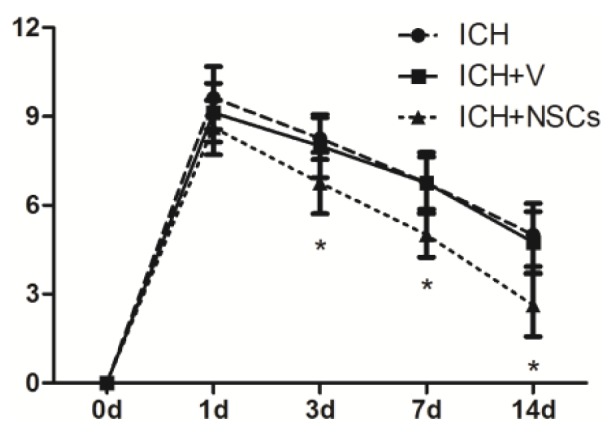
Behavioral functional test before and after ICH. Transplanted NSCs improve functional recovery after ICH at day 3, 7 and 14. (*n* = 6 in each group) * *p* < 0.05 *versus* ICH and ICH + V.

For rats treated with NSCs at day 1 after ICH, no significant difference in functional recovery was found. However, transplanted NSCs improve neurological function recovery at day 3, 7 and 14 compared with ICH and ICH-vehicle groups (Figure 3).

### 2.4. Transplanted NSCs Modulate γδT and Treg Cells after ICH

**Figure 4.**
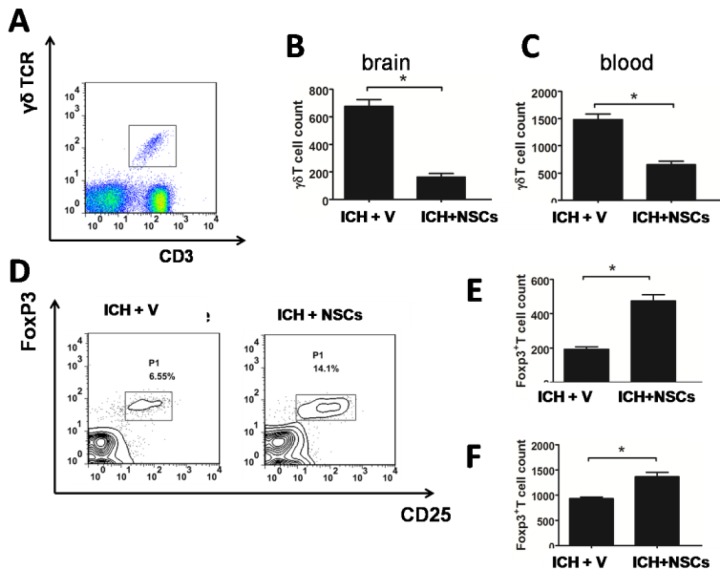
Transplanted NSCs altered γδT and Treg cells in the brain and peripheral blood. Transplanted NSCs reduced the absolute numbers of γδT cells in the brain and peripheral blood (**A**–**C**); The percentage and the absolute numbers of Treg cells were significantly increased in the brain by transplantation of NSCs (**D**,**E**); An increased absolute Treg cell numbers in the peripheral blood after transplanted NSCs (**F**). (*n* = 8 in each group) * *p* < 0.05 *versus* ICH-vehicle.

Next, we determined whether transplanted NSCs affected the number of γδT and Treg cells at 3 days after ICH. As shown in Figure 4A,B, the absolute number of γδT lymphocytes in the brain was significantly decreased 3 days after ICH and NSC transplantation. Similar finding was observed in the peripheral blood (Figure 4C). In addition, absolute number of Treg cells in the brain as well as in the peripheral blood in NSC-treated group was also greater than compared with vehicle-treated group after ICH (Figure 4D–F).

### 2.5. Transplanted NSCs Altered Cytokines in the Cerebral and Peripheral Blood

**Figure 5.**
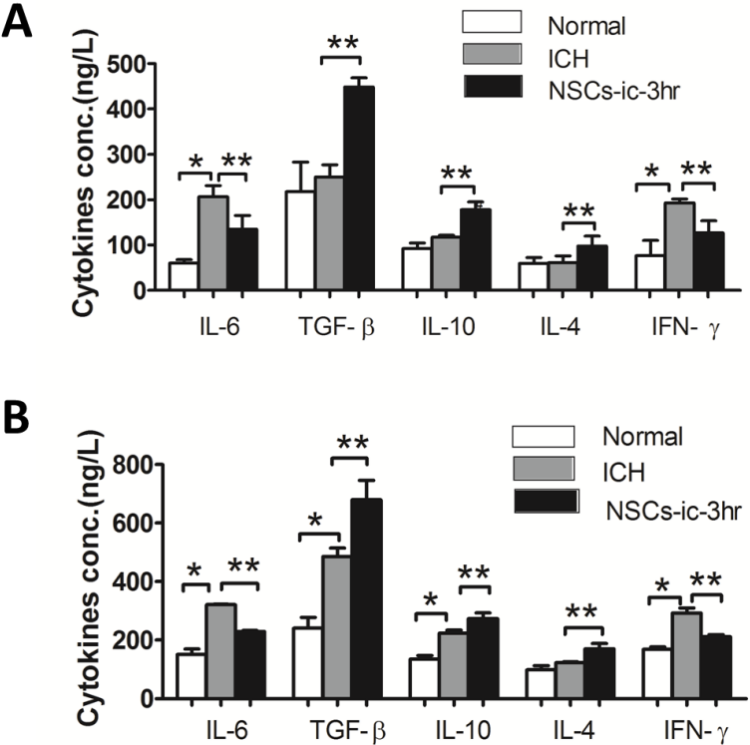
Expressions of inflammatory cytokine in the cerebral and peripheral blood after transplantation of NSCs. ELASA (**A**,**B**) revealed the upregulations of IL-6 and IFN-γ in the haemorrhagic brains and peripheral blood in the groups transplanted witn NSCs, compared to the normal samples. In the peripheral blood, TGF-β and IL-10 were upregulated after ICH followed NSC transplantation. However, IL-4 was unchanged both in the brain and peripheral blood after ICH. In brain and peripheral blood samples, the groups treated with NSCs showed decreases of IL-6 and IFN-γ and increases of IL-4, IL-10 and TGF-β levels, compared with the ICH group. (*n* = 4 in each group) * *p* < 0.05 *versus* normal; ** *p* < 0.05 *versus* ICH.

Finally, we investigated the levels of cytokines in the brain and peripheral blood 3 days after ICH followed NSC transplantation. As shown in Figure 5, IL-6 and IFN-γ were upregulated in the hemorrhagic brains and peripheral blood, compared to normal samples. TGF-β and IL-10 were increased in the peripheral blood. While IL-4 was unchanged both in the peripheral blood and brain after ICH.

The rats treated with NSCs showed decreases of IL-6 and IFN-γ levels, and increases of TGF-β, IL-10, and IL-4 levels in brain and peripheral blood, compared with vehicle-treated groups (Figure 5A,B).

## 3. Discussion

In this study, we investigated the immunomodulation effects of transplanted NSCs on T lymphocyte. We found that intracerebral transplantation of NSCs reduced detrimental γδT cells, and increased protective Treg cells in the hemorrhagic brain day 3 after ICH. Similar findings were also observed in the peripheral blood. In addition, we also observed that transplanted NSCs exhibit anti-inflammatory properties with decreased pro-inflammatory cytokines and increased anti-inflammatory cytokines in the brain and peripheral blood.

Numerous studies have demonstrated that the inflammatory responses play a critical role in post-stroke brain injury and the extent of neuronal damage seemed to correlate with the degree of immune activity [23]. The crucial functions of invading T cells and inflammatory cytokines have been well studied in ischemic stroke [8,9], but evidence with regard to T cell infiltration after ICH is largely unexplored. One study showed that CD4^+^T cells infiltrated into brain 4 days after ICH [7]. The harmful effects of γδT cells and beneficial effects of Treg cells in ischemic stroke have been well documented. Our study confirmed that γδT and Treg cells were altered in the brain after ICH.

To date, it has been demonstrated that transplantation of NSCs promoting post-ICH functional recovery mainly depends on replacing lost neurons and establishing neuron-neuron network [16,24]. However, substantial evidence recently suggested that transplanted NSCs-mediated recovery of neurological functions may be due to immunomodulatory mechanisms rather than direct cell replacement. In the EAE model, transplanted NSCs are able to promote neuroprotection by exerting unexpected immune-like functions, such as suppressing T cells activity and inducing T cells apoptosis [17]. In addition, *in vitro* NSCs and T cells co-culture studies suggested that NSCs block T cells proliferation through secreted mediator(s) or contact inhibition [20,21,25]. Therefore, our present study is the first to report that earlier intracerebral NSCs administration during the hyperacute stage in ICH can modulate cerebral and circulation immune responses by interacting with T lymphocyte subtypes.

As observed by this study, different T cells traffic into the brain parenchyma after ICH and earlier cerebral transplanted NSCs can modulate the number of infiltrating T cells. There are two potential approaches that T cells enter into the brain parenchyma after ICH. Some T cells migrate from the hematoma into the perihematomal tissue and others may infiltrate through the opened BBB [26,27]. As previous reports [16], intracerebral transplanted NSCs in the core of the hematoma migrated to the border of the hematoma. Therefore, we can hypothesize that NSCs may repair the damaged BBB directly or inhibit transendothelial migration of detrimental T lymphocyte subpopulations via contact inhibition. Transplanted NSCs can also promote protective T cells infiltration through release of chemotactic factors. Taken together, these mechanisms involving transplanted NSCs altered T cell subpopulations infiltrating to the brain parenchyma after ICH. It has been known that the central nervous, neuroendocrine, and the immune systems interact with each other. Transplanted NSCs can alter cerebral milieu after ICH such as cytokines, immunomodulatory substances, neurotrophic growth factors, which can activate neuroendocrine pathways like the hypothalamic-pituitary-adrenal (HPA) axis and the sympathetic nervous system [28,29]. These two activated systems’ subsequent release of cortisol and catecholamines modulate the immune system and resulted in alteration of T cell subpopulation counts in the peripheral blood.

Inflammatory cytokines are secreted by diverse cells and have deleterious or beneficial roles in stroke. Previous studies have shown that T cells are the major source of IFN-γ in the ischemic brain and IFN-γ antagonization substantially reduce infarct size [8,30,31]. TGF-β produced by neurons, microglia and macrophages can also suppress inflammation by inhibiting the response of T helper cells and promoting the development of Treg cells [32]. Similarly, IL-10 secreted by multiple cells including Treg cells has both neuroprotective and anti-inflammatory effects [30,33]. In this study, we demonstrated that transplanted NSCs attenuate IFN-γ expression and increase TGF-β and IL-10 expression. These results not only elucidate anti-inflammatory effects of NSCs, but also partially reflect T cell subpopulations alteration in the brain and blood after ICH. NSCs transplantation also upregulated IL-4, an anti-inflammatory molecule produced by T helper 1 cells [34].

In conclusion, our study provides new insights into early transplantation of NSCs modulate immune response after acute ICH. Specifically, we have described a previously unknown role of transplanted NSCs as immunomodulators after ICH, a function that affects different T cell subpopulations expressed in the brain and peripheral blood after ICH.

## 4. Experimental Procedure

### 4.1. ICH Model

The animal experiment was conducted in accordance with national guidelines for the use of experimental animals and the protocols were approved by the Animal Care Committee of the Wenzhou Medical University (Wenzhou, China). A total of ninety four (*n* = 94) adult male Sprague-Dawley (SD) rats (280–320 g) were used in all our experiments. ICH was induced via stereotaxic, intrastriatal administration of bacterial collagenase IV (0.25 IU dissolved in 1 μL saline, Sigma, St. Louis, MO, USA) using the protocol as described previously [35,36]. In brief, rats underwent intraperitoneal injection of 10% chloral hydrate (0.4 mL/100 g) and placed in a stereotaxic frame (Kopf Instruments, Tujunga, CA, USA). A midline skin incision was made to the scalp to expose the skull and bregma. A burr hole was made and a 30-gauge needle was inserted into the striatum according to the coordinates (0.2 mm posterior, 6.0 mm ventral, and 3.0 mm lateral to the bregma). Collagenase IV was administrated over a period of 5 min. The needle was left for another 5 min and then removed gently. The burr hole was filled with bone wax, and the incision was sutured. Sham ICH was induced with an injection of equal volume (1 μL) of saline instead of collagenase. Animals were maintained in separate cages at room temperature, 25 °C, with free access to food and water under a 12-h light-dark cycle. In this experiment, rats that lacked of neurological deficit were excluded.

### 4.2. Cell Preparation and Transplantation Procedure

NSCs (C57BL/6 Mouse NSCs) were purchased from Cyagen Biosciences, US (No. MUBNF-01001). These primary stem cells were obtained from the hippocampus and subependymal zone of the fetal brain of 12.5-day gestational age of C57BL/6 mice, cultured in mouse NSCs complete medium, passaged and amplified to the first generation, and stored frozen. NSCs were thawed and transferred to tubes containing the medium, and centrifuged at 1000 rpm for 5 min. Cells were added with 2–3 mL medium, dispersed gently and mixed fully after removing the supernatant. The cell suspension was transferred to a 25 cm^2^ flask, added with the sufficient medium, and incubated at 37 °C, 5% CO_2_. Depending on the rate of cell growth and the change of medium color, we performed passage culture at a 3–4 day interval.

Three hours after ICH induction, behavioral tests were performed and rats without neurological deficits were excluded. NSCs (5 × 10^5^ cells in 2 μL) or vehicle (2 μL of PBS) was intracerebrally injected into the brain via the primary burr hole at 3 h after ICH.

### 4.3. Behavioral Testing

For behavioral test, rats were tested before ICH and at 1, 3, 7, and 14 days after ICH and transplantation of NSCs by an investigator who was blinded to the experimental groups using a set of modified Neurological Severity Scores (mNSS) [37]. mNSS is a complex behavioral test including motor, sensory, reflex and balance tests. In this test, the score ranges from 0 to 18, where 0 is considered normal and 18 is maximum deficit.

### 4.4. Isolation of Brain and Blood Lymphocytes

Rats were anesthetized and transcardially perfused with 200 mL normal saline, and the brains were removed from the skull immediately. Two hemispheres were put together mechanically dissociated and incubated for 30 min at 37 °C in dissociation buffer (1 mg/mL collagenase, 0.1 mg/mL DNase I in DMEM), and pressed through a 40-μm nylon cell strainer (Becton Dickinson, Franklin Lakes, NJ, USA). The cell suspension was collected and centrifuged at 1200 rpm for 5 min at room temperature; the pellet was resuspended in 4 mL of 30% Percoll (GE Healthcare, Uppsala, Sweden) and overlaid on the top of 37% and 70% Percoll solution. The gradient was centrifuged at 500× *g* for 20 min at room temperature and cells were collected on the interface between 37% and 70% Percoll. Isolated cells were washed once (1200 rpm, 5 min) with 10 mL PBS and were resuspended in 100 μL of flow cytometry staining buffer (eBioscience, San Diego, CA, USA).

Venous blood (1 mL each group) was collected and lymphocytes were isolated according to the manufacturer’s instruction (eBioscience). Flow cytometry staining buffer (100 μL) was used to resuspend the isolated cells for flow cytometry analysis.

### 4.5. Flow Cytometric Analysis

Cells isolated from the CNS and peripheral blood were stained for anti-CD3APC, anti-CD4FITC, anti-γδTCRPE, anti-CD25FITC, anti-Foxp3PE, and the appropriate isotype control respectively according to the manufacturer’s protocols (eBioscience, San Diego, CA, USA). Flow cytometry was performed using a Becton Dickinson FACS Calibur and the data were analysed by CellQuest Pro software (BD Bioscience, Franklin Lakes, NJ, USA). Gates were set according to unstained samples and isotype control; compensation was adjusted using BD CaliBRITE Beads (BD Bioscience, Franklin Lakes, NJ, USA). In the blood, we counted 100,000 cells of all samples.

### 4.6. Cytokine Enzyme-Linked Immunosorbent Assay

Serum was collected and frozen immediately until analysis of cytokine protein levels with commercial kits. The ipsilateral striatal tissue (100 mg) were collected and protein was isolated. The collected serum and brain tissue samples were analyzed for IL-4, IL-6, IL-10, TGF-β1 and IFN-γ using enzyme-linked immunosorbent assay (R&D Systems, Indianapolis, MN, USA).

### 4.7. Statistical Analysis

The data were presented as mean ± standard deviation (SD). All values were statistically analyzed using Prism software for unpaired Student’s *t*-test. Data comparing multiple groups were analyzed using one-way ANOVA. A *p* value of <0.05 is considered statistically significant.

## 5. Conclusions

In summary, our study suggest that immunomodulation effect of neuroprotection via early injection of intracerebral NSCs after ICH is through increasing protective T cells and anti-inflammatory cytokine secretion, meanwhile reducing detrimental T cells and pro-inflammatory cytokines secretion.
